# Serum Phthalate and Triclosan Levels Have Opposing Associations With Risk Factors for Gestational Diabetes Mellitus

**DOI:** 10.3389/fendo.2018.00099

**Published:** 2018-03-13

**Authors:** Benjamin G. Fisher, Hanne Frederiksen, Anna-Maria Andersson, Anders Juul, Ajay Thankamony, Ken K. Ong, David B. Dunger, Ieuan A. Hughes, Carlo L. Acerini

**Affiliations:** ^1^Department of Paediatrics, University of Cambridge, Cambridge, United Kingdom; ^2^EDMaRC, Department of Growth and Reproduction, Rigshospitalet, University of Copenhagen, Copenhagen, Denmark; ^3^MRC Epidemiology Unit, Institute of Metabolic Science, University of Cambridge, Cambridge, United Kingdom

**Keywords:** gestational diabetes, pregnancy, phthalates, bisphenol A, triclosan, endocrine disrupting chemicals, environmental chemicals

## Abstract

Certain phthalates and bisphenol A (BPA) have been associated with insulin resistance and type 2 diabetes in non-pregnant adults, but studies of gestational diabetes mellitus (GDM) have reported conflicting results for phthalates and no associations with BPA. Our aim was to investigate the relationship between maternal serum levels of phthalate metabolites and phenols at 10–17 weeks of gestation and glucose homeostasis at 28 weeks of gestation. 232 women aged ≥16 years without type 1 or 2 diabetes with singleton male pregnancies were recruited from a single UK maternity centre between 2001 and 2009 as part of a prospective observational study (Cambridge Baby Growth Study). Serum levels of 16 phthalate metabolites and 9 phenols (including BPA) were measured using liquid chromatography/tandem mass spectrometry. Oral glucose tolerance tests were performed at 28 weeks. 47/232 (20.3%) women had GDM. First-trimester triclosan (TCS) was inversely associated with incident GDM (adjusted odds ratio per log increase in concentration 0.54, 95% confidence interval 0.34–0.86, *p* = 0.010). Amongst women without GDM, first-trimester mono-(2-ethylhexyl) phthalate and mono(carboxyisooctyl) phthalate levels were positively associated with 120-min plasma glucose (adjusted β 0.268 and 0.183, *p* = 0.0002 and 0.010, respectively) in mid-pregnancy. No other monotonic associations were detected between phthalate or phenol levels and fasting or stimulated plasma glucose, β-cell function, insulin resistance, or 60-min disposition index. Our results support a glycaemia-raising effect of phthalates during pregnancy, consistent with findings in non-pregnant populations and suggest a possible protective effect of exposure to TCS against GDM.

## Introduction

Late pregnancy is characterised by peripheral insulin resistance with a compensatory increase in insulin secretion and β-cell mass. Inadequacy of this response leads to gestational diabetes mellitus (GDM), which is associated with both perinatal and long-term adverse outcomes (1). The incidence of GDM in developed countries has increased significantly in recent years, a trend that cannot be fully explained by rising levels of maternal obesity (2). There is increasing interest in a possible role for endocrine disrupting chemicals (EDCs), exogenous substances that interfere with the endocrine system and cause adverse health effects. Two groups of EDCs that have been well studied in the context of glycaemia are phthalic acid esters (phthalates) and bisphenol A (BPA, 4,4′-dihydroxy-2,2-diphenylpropane).

Phthalates are ubiquitous industrial chemicals, several metabolites of which are detectable in the urine of >75% of the general population (3). *In vitro*, phthalates activate peroxisome proliferator-activated receptors, which regulate cellular lipid and glucose metabolism (4), and reduce insulin receptor concentration (5). In rodents, phthalates impair insulin secretion and glucose tolerance (6). Epidemiological studies in non-pregnant adults have demonstrated associations between phthalates and raised fasting blood glucose, insulin resistance, reduced insulin secretion, and type 2 diabetes mellitus (T2DM) (7–9). To date, only three epidemiological studies have assessed the associations between phthalates and gestational glycaemia. In a cohort of 72 women, first-trimester urinary concentrations of monoisobutyl phthalate (MiBP) and monobenzyl phthalate (MBzP) were inversely associated with 60-min blood glucose levels measured 3–25 weeks later (10). In a larger study (*n* = 350), second-trimester urinary monoethyl phthalate (MEP) was positively associated with impaired glucose tolerance (IGT), whereas di-(2-ethylhexyl) phthalate (DEHP) metabolites were associated with lower odds of IGT (11). The largest study (*n* = 1,274) observed no significant associations between first-trimester urinary phthalate metabolites and incident IGT/GDM (12).

Bisphenol A is another commonly used industrial chemical, detectable in the urine of 93% of the US population (13). It exerts oestrogenic and various other effects (7). In rodents, BPA causes disrupted insulin signalling, insulin resistance, and IGT (14). Epidemiological studies in non-pregnant adults have generated conflicting results for associations between BPA and insulin resistance and T2DM (9, 15–17). In the three studies of pregnant women to date, only one prospective cohort study (*n* = 245) reported a positive association between second-trimester (but not first-trimester) urinary BPA levels and stimulated blood glucose levels (but not incident GDM) at 24–28 weeks of gestation (18). In a larger study (*n* = 1,274), first-trimester urinary BPA was not significantly associated with incident IGT/GDM (12), and in a small case-control study (*n* = 94), second-trimester urinary BPA was not associated with GDM or fasting blood glucose levels amongst controls (19).

Other phenol compounds [4-tert-octyl phenol, benzophenone-3 (BP-3), and triclosan (TCS)] have not been associated with T2DM or fasting serum glucose in humans (20).

Our objective was to investigate the prospective relationships between maternal serum levels of phthalate metabolites, BPA, and other phenols at 10–17 weeks of gestation and glucose homeostasis at 28 weeks of gestation in a UK setting.

## Materials and Methods

### Study Design

Pregnant women aged ≥16 years (*n* = 2,229) were recruited to the Cambridge Baby Growth Study (CBGS), a large prospective cohort study, from routine antenatal clinics at 10–17 weeks of gestation at the Rosie Maternity Unit, Cambridge, between April 2001 and March 2009. Characteristics of CBGS participants have previously been described (21). Written informed consent was obtained from all women.

A subcohort of 330 mothers of 334 male infants was selected for a nested case-control study of EDCs and cryptorchidism (30 infants with congenital cryptorchidism, 21 infants with postnatally acquired cryptorchidism, 285 controls). Of these, women were retrospectively included in the present study if they had a singleton pregnancy, no pre-existing diagnosis of type 1 or 2 diabetes mellitus or GDM, and undertook an oral glucose tolerance test (OGTT) at 28 weeks of gestation. This subset of 232 women was representative of the whole cohort with regard to age, ethnicity, pre-pregnancy body mass index (BMI), and deprivation index (all *p* > 0.2).

Non-fasting maternal serum samples were collected into plastic-free glass tubes at enrolment for measurement of 16 phthalate metabolites and 9 phenols (Table 1). Serum samples were stored at −70°C. OGTTs were performed to diagnose GDM and assess β-cell function and insulin resistance.

**Table 1:** Medians, quartiles, and ranges of serum concentrations of the investigated phthalate metabolites and phenols in the study population (*n* = 232 for phthalates, *n* = 228 for phenols).

Chemical concentration (μg/l)^a^	Limit of detection (LOD)	Detection rate (%)	Minimum	Percentiles			Maximum
				25th	50th	75th	
**Phthalate metabolites [parent compound]**							
Monoethyl phthalate (MEP) [DEP]^b^	0.50	90.5	<LOD	0.79	1.56	4.26	172.05
Monoisobutyl phthalate (MiBP) [DiBP]^b^	0.77	97.8	<LOD	1.97	3.78	6.60	29.34
Mono-n-butyl phthalate (MnBP) [DnBP]^b^	0.82	71.6		<LOD	1.34	2.31	9.41
Mono-pentyl phthalate (MPP) [DPP]^b^	0.20	0.4				<LOD	0.24
Monobenzyl phthalate (MBzP) [BBzP]^b^	0.40	6.9				<LOD	1.08
Mono-(2-ethylhexyl) phthalate (MEHP) [DEHP]^b^	0.60	63.8		<LOD	1.14	58.53	421.93
Mono-(2-ethyl-5-hydroxyhexyl) phthalate (MEHHP) [DEHP]	0.56	6.9				<LOD	4.45
Mono-(2-ethyl-5-oxohexyl) phthalate (MEOHP) [DEHP]	0.35	2.3				<LOD	1.94
Mono-(2-ethyl-5-carboxypentyl) phthalate (MECPP) [DEHP]	0.28	79.3	<LOD	0.30	0.52	0.95	14.39
Mono-n-octyl phthalate (MOP) [DnOP]^b^	0.20	0.4				<LOD	0.26
Mono-(3-carboxypropyl) phthalate (MCPP) [DnOP]	0.36	0.4				<LOD	0.44
Mono-isononyl phthalate (MiNP) [DiNP]^b^	0.20	19.0				<LOD	3.24
Mono(hydroxyisononyl) phthalate (MHiNP) [DiNP]	0.38	0.0					<LOD
Mono-oxoisononyl phthalate (MOiNP) [DiNP]	0.10	3.4				<LOD	0.22
Mono(carboxyisooctyl) phthalate (MCiOP) [DiNP]	0.10	75.4	<LOD	0.10	0.18	0.37	13.76
Mono-isodecyl phthalate (MiDP) [DiDP]^b^	0.20	3.4				<LOD	0.43
**Phenols**							
Bisphenol A (BPA)	0.18	89.9	<LOD	1.02	1.76	2.96	8.46
Triclosan (TCS)	0.22	69.3		<LOD	0.93	8.29	178.75
Triclocarban (TCC)	0.27	26.3			<LOD	0.36	18.85
Benzophenone-3 (BP-3)	0.12	70.2		<LOD	0.34	0.79	67.11
2,4-dichlorophenol (2,4-DCP)	0.18	11.4				<LOD	0.69
2,5-dichlorophenol (2,5-DCP)	0.11	4.5				<LOD	3.88
2,4,5-trichlorophenol (2,4,5-TCP)	0.29	0.4				<LOD	1.26
2-phenylphenol (2-PP)	0.13	41.2			<LOD	0.23	0.74
4-phenylphenol (4-PP)	0.12	5.7				<LOD	0.37

*^a^<LOD: below the LOD*.

*^b^Semiquantitative data (see Materials and Methods)*.

### Maternal Demographic Characteristics

A questionnaire administered at ~36 weeks of gestation collected demographic information, including history of diabetes mellitus, ethnicity, smoking, and parity. The Index of Multiple Deprivation 2007 (IMD), which measures various economic, social, and housing parameters, was derived from residential postcodes (22). The sample mean IMD value (9.37) was imputed for those cases where it was missing (*n* = 54) as these women did not differ from others with regard to age, pre-pregnancy BMI, ethnicity, smoking status, parity, or prevalence of GDM (all *p* > 0.3).

### Oral Glucose Tolerance Testing

At 28 weeks of gestation, a 75-g OGTT was performed following an overnight fast. Venous whole blood glucose levels were measured at 0 and 60 min, and capillary blood glucose levels were measured at 0, 60, and 120 min. From May 2007 onwards, venous whole blood glucose levels were also measured at 120 min (in 56/232 women, 24.1%). Women completing OGTTs before May 2007 did not differ from those completing OGTTs during/after May 2007 with regard to age, BMI, IMD, ethnicity, smoking status, parity, or prevalence of GDM (all *p* > 0.1). Insulin and C-peptide levels were measured at 0 and 60 min in 158/232 (68.1%) women. Women with missing insulin and C-peptide levels did not differ from others with regard to age, BMI, IMD, ethnicity, smoking status, parity, or prevalence of GDM (all *p* > 0.1). Glycated haemoglobin levels were not measured.

### Assays

We measured phthalate metabolites rather than their diester parent compounds, as the latter are metabolised rapidly (23), and exposure misclassification could potentially occur from contamination of the samples with phthalate diesters during venesection or laboratory handling. Serum samples were analysed for the total content of 16 phthalate metabolites (from 9 parent phthalate diesters) and 9 phenols (Table 1) by isotope-diluted liquid chromatography coupled to tandem mass spectrometry. The method and determination of the limit of detection (LOD) was as previously described (24, 25) (Supplementary Methods in Supplementary Material). Measured levels of the phthalate monoesters were considered to be semiquantitative, as hydrolysis of potentially contaminating diester phthalates to monoester phthalates [e.g., DEHP to mono-(2-ethylhexyl) phthalate (MEHP)] by enzymes present in blood may have occurred after the samples were drawn. However, the large interindividual variation observed for these metabolites suggests that a systematic contamination of the samples with one or more of the phthalate diesters is unlikely. The measured levels for downstream phthalate metabolites [mono-(2-ethyl-5-hydroxyhexyl) phthalate (MEHHP), mono-(2-ethyl-5-oxohexyl) phthalate, mono-(2-ethyl-5-carboxypentyl) phthalate (MECPP), mono-(3-carboxypropyl) phthalate, mono(hydroxyisononyl) phthalate, mono-oxoisononyl phthalate, mono(carboxyisooctyl) phthalate (MCiOP)] were quantitative, as conversion to these metabolites can occur only *in vivo*.

Whole blood glucose levels were measured using a standard glucose oxidase-based assay. Capillary blood glucose measurements were made using an Abbott Freestyle Mini (Abbott Diagnostics, Maidenhead, UK). Fasting and stimulated insulin and C-peptide levels were measured by enzyme-linked immunosorbent assay using commercial kits (DSL, London, UK).

### Calculations

Analyses of associations with parameters of blood glucose homeostasis included only those chemicals measured above the LOD in >60% of samples. Concentrations below the LOD were assigned a value equal to LOD/2 if the data were highly skewed or LOD/√2 if not (26). These substituted data were then used for all subsequent analyses. Concentrations were log-transformed to correct for non-normal distributions with positive skew. For categorical analyses, subjects were categorised into quartiles using the 25th, 50th, and 75th percentiles of the subcohort (*n* = 232), with the first quartile used as the referent category.

For the purposes of diagnosing GDM and estimating β-cell function and insulin resistance, venous plasma glucose levels were estimated by multiplying venous whole blood glucose levels, where available, by 1.08, assuming a haematocrit of 31.5% (27, 28). For the 176 women who completed an OGTT prior to May 2007 and, therefore, did not have a 120-min venous whole blood glucose level measured, 120-min venous plasma glucose level was estimated using the equation venous plasma glucose = 1.035 × capillary blood glucose—0.891, which was derived from a linear regression model assessing the ability of 60-min capillary blood glucose to predict 60-min venous plasma glucose using data from the entire CBGS cohort [*R*^2^ = 0.790, *F*(1,768) = 2,889.3, *p* = 2 × 10^−262^]. A linear regression model assessing the ability of fasting capillary blood glucose to predict fasting venous plasma glucose gave a different result: venous plasma glucose = 0.826 × capillary blood glucose + 0.996 [*R*^2^ = 0.511, *F*(1,759) = 792.6, *p* = 6 × 10^−120^]; however, we justified using the first equation to estimate the missing 120-min venous plasma glucose levels as across the entire CBGS cohort the mean 60-min venous plasma glucose (7.4 mmol/l) was closer to the mean 120-min venous plasma glucose (6.9 mmol/l) than the mean fasting venous plasma glucose (4.7 mmol/l) The updated homeostasis model assessment (HOMA2) was used to estimate steady-state β-cell function (HOMA2-B, using fasting C-peptide and plasma glucose levels) and insulin resistance (HOMA2-IR, using fasting insulin and plasma glucose levels) (29); software for calculating these values is available online (https://www.dtu.ox.ac.uk/homacalculator/). To estimate insulin secretion independent of insulin sensitivity, we calculated the disposition index as the 60-min insulinogenic index [(insulin 60 − insulin 0)/(venous glucose 60 − venous glucose 0)] divided by HOMA2-IR (30). HOMA2-B, HOMA2-IR, and disposition index were log-transformed to normalise distributions.

### Diagnosis of GDM

For primary analyses, women were diagnosed with GDM if they met one or more of the following criteria: fasting plasma glucose ≥5.1 mmol/l, 60-min plasma glucose ≥10.0 mmol/l, or 120-min plasma glucose ≥8.5 mmol/l (31, 32). For sensitivity analyses, the following criteria were used: fasting plasma glucose ≥7.0 mmol/l or 120-min plasma glucose ≥7.8 mmol/l (33).

### Neonatal Examinations

Newborns were examined by research nurses, as far as possible in the first 2 weeks of life, either in hospital or at home visits. They were then re-examined during clinic visits at 3, 12, and 24 months of age. Birth weight as measured at delivery by midwives was taken from health records. Cryptorchidism was defined as one or more testes not present in the inferior half of the scrotum for at least a few moments after manipulation.

### Ethical Approval

The study was approved by Cambridge Local Research Ethics Committee and adhered to the Declaration of Helsinki.

### Statistical Analyses

Distribution percentiles of phthalate and phenol concentrations were calculated for women with and without GDM, and differences in patient characteristics were assessed using the *t*-test and Mann–Whitney *U*-test for continuous variables, and chi-square test for categorical variables.

Logistic regression was used to model the odds of GDM in relation to serum phthalate/phenol concentrations, adjusting for covariates of interest. Continuous relationships between phthalate/phenol levels and HOMA2-B, HOMA2-IR, fasting and 120-min plasma glucose, and disposition index were individually assessed using multiple linear regression, adjusting for covariates of interest. We restricted these latter analyses to women who did not have GDM, because in women with GDM glucose toxicity *per se* may cause insulin resistance and β-cell dysfunction (8). Outliers (data points with standardised residuals >3SDs) were removed in all regressions (typically 0–2 cases per regression). Tests of heterogeneity (*p*_het_) and linear trend (*p*_trend_) in outcome value across serum phthalate/phenol concentration quartiles were performed. Because numerous chemicals were significantly correlated and several have been previously associated with dysglycaemia (7–11, 15–17), regression analyses were conducted separately for each chemical, and we did not correct for multiple testing.

The following potential confounders were chosen *a priori* as covariates in the multivariable analyses because of their known biological relevance to the outcomes of interest (12): age, pre-pregnancy BMI (log-transformed to normalise the distribution), IMD (log-transformed), and parity (nulliparous vs. para 1 vs. para 2 or greater). We did not use ethnicity or smoking status in our regressions, because very few women were of non-white ethnicity (4/151, 2.6%) or were smokers (5/232, 2.2%), and data on ethnicity were missing for 81/232 (34.9%) women.

In view of the nature of our subcohort (case-control study of cryptorchidism) and a possible association between GDM and congenital cryptorchidism (34), we additionally used logistic regression to model the odds of GDM in relation to cryptorchidism at birth, and multiple linear regression to assess the relationship between cryptorchidism and HOMA2-B, HOMA2-IR, fasting/stimulated plasma glucose, and disposition index in women who did not have GDM, adjusting all analyses for the above covariates as well as prematurity (gestation <37 vs. ≥37 weeks) and small for gestational age (birth weight SD score <−1.5 vs. ≥−1.5), which are both risk factors for cryptorchidism (34). Finally, we used logistic regression to remodel the odds of GDM in relation to serum phthalate/phenol concentrations after excluding mothers of sons with congenital cryptorchidism.

All *p*-values were two-sided and *p* < 0.05 was considered statistically significant. Data were analysed using SPSS, version 22.0 (IBM Corporation, New York, NY, USA).

## Results

232 women participated in the study, of whom 47 (20.3%) met one or more criteria for GDM (Table 2). Of these, 34 (72.3%) had an abnormal fasting plasma glucose, 18 (38.3%) had an abnormal 60-min glucose, and 14 (29.8%) had an abnormal 120-min glucose.

**Table 2:** Characteristics of the study population.

Characteristics^a^	Women with gestational diabetes mellitus (GDM) (*n* = 47)	Women without GDM (*n* = 185)	*p-*Value for GDM vs. no GDM^b^
Age (years)	33.1 ± 4.4	33.7 ± 3.8	0.30
Pre-pregnancy body mass index (kg/m^2^)	25.4 ± 5.1	23.7 ± 3.7	0.051
**Ethnicity^c^**			
White	28 (100%)	119 (96.7%)	0.75
Other	0 (0%)	4 (3.3%)	
**Current smoker**			
No	46 (97.9%)	181 (97.8%)	0.99
Yes	1 (2.1%)	4 (2.2%)	
**Parity**			
0	21 (44.7%)	87 (47.3%)	0.91
1	20 (42.6%)	72 (39.1%)	
≥2	6 (12.8%)	25 (13.6%)	
Index of multiple deprivation (U)	9.43 ± 3.48	9.35 ± 4.27	0.90
Fasting plasma glucose (mmol/l)	5.5 ± 0.9	4.5 ± 0.3	4 × 10^−9^*
60-min plasma glucose (mmol/l)	9.5 ± 2.0	7.1 ± 1.5	1 × 10^−10^*
120-min plasma glucose (mmol/l)	7.6 ± 1.7	5.9 ± 1.0	3 × 10^−8^*
HOMA2-B (%)	113.7 (94.7–125.9)	120.0 (106.0–146.1)	0.07
HOMA2-IR (U)	1.35 (0.96–1.68)	0.82 (0.67–1.08)	2 × 10^−6^*
Disposition index (mmol/pmol.U)	119.7 (59.6–160.2)	152.7 (112.0–284.9)	0.001*
**Infant with congenital cryptorchidism**			
No	43 (91.5%)	173 (93.5%)	0.87
Yes	4 (8.5%)	12 (6.5%)	

*^a^Mean ± SD or median (IQR) for continuous variables, *n* (%) for categorical variables*.

*^b^Mann–Whitney *U*-test for HOMA2-B, HOMA2-IR, and disposition index; *t*-test for other continuous variables; chi-square test for categorical variables (with continuity correction for 2 × 2 tables)*.

*^c^Data on ethnicity are missing for n = 81 women*.

*p < 0.05.

Six phthalate metabolites [MEP, MiBP, mono-n-butyl phthalate (MnBP), MEHP, MECPP, MCiOP] and three phenols (BPA, triclosan, BP-3) were detectable in >60% of samples (Table 1; Table S1 in Supplementary Material). Some phthalate metabolites were strongly or moderately intercorrelated (MnBP and MiBP, rho = 0.838, *p* = 2 × 10^−62^; MECPP and MCiOP, rho = 0.357, *p* = 2 × 10^−8^), and several of the other chemicals were weakly correlated (Table S1 in Supplementary Material).

In continuous models, of these nine chemicals, only triclosan was associated with incident GDM [adjusted OR (aOR) per log increase in concentration 0.54, 95% CI 0.34–0.86, *p* = 0.010] (Table 3). We conducted a categorical analysis to look for threshold and non-linear effects (Table 4). In accordance with the continuous analysis, this demonstrated lower odds of GDM in the second (aOR 0.25, 95% CI 0.07–0.86, *p* = 0.028), third (aOR 0.12, 95% CI 0.03–0.55, *p* = 0.007), and fourth (aOR 0.35, 95% CI 0.12–0.98, *p* = 0.046) quartiles for triclosan, compared to the first quartile (*p*_het_ = 0.009, *p*_trend_ = 0.022). Inspection of the data did not reveal evidence for a threshold or non-linear relationship. Conversely, women with MiBP levels in the second (aOR 5.69, 95% CI 1.56–20.73, *p* = 0.008) and fourth (aOR 4.89, 95% CI 1.32–18.14, *p* = 0.018) quartiles, but not in the third quartile, had higher odds of GDM (*p*_het_ = 0.001, *p*_trend_ = 0.41).

**Table 3:** Adjusted odd ratios and 95% CIs for incident gestational diabetes mellitus in relation to log-transformed serum chemical concentrations.

Log-transformed serum chemical concentration	Adjusted model^a^	
	OR (95% CI)	*p-*Value
Monoethyl phthalate	0.81 (0.39–1.70)	0.58
Monoisobutyl phthalate	1.48 (0.51–4.34)	0.47
Mono-n-butyl phthalate	1.55 (0.45–5.33)	0.49
Mono-(2-ethylhexyl) phthalate	0.93 (0.66–1.31)	0.67
Mono-(2-ethyl-5-carboxypentyl) phthalate	0.75 (0.27–2.06)	0.57
Mono(carboxyisooctyl) phthalate	1.12 (0.47–2.66)	0.81
Bisphenol A	1.16 (0.48–2.78)	0.74
Triclosan	0.54 (0.34–0.86)	0.010*
Benzophenone-3	0.80 (0.44–1.44)	0.45

*^a^Adjusted for age, pre-pregnancy body mass index (log-transformed), IMD (log-transformed), and parity; *n* = 197 for phthalates, *n* = 193 for phenols*.

*p < 0.05.

**Table 4:** Adjusted odd ratios and 95% CIs of incident gestational diabetes mellitus (GDM) by category of concentrations of the different chemicals.

Chemical	Quartile 1	Quartile 2	Quartile 3	Quartile 4	*p-*Value for heterogeneity	*p*-Value for linear trend
Monoethyl phthalate						
Incidence of GDM (%)	10/58 (17.2%)	14/58 (24.1%)	10/58 (17.2%)	13/58 (22.4%)	0.45	0.87
Adjusted OR (95% CI)^a^	Referent	1.65 (0.60–4.56)	0.67 (0.21–2.07)	1.19 (0.42–3.37)		

Monoisobutyl phthalate						
Incidence of GDM (%)	9/58 (15.5%)	17/58 (29.3%)	6/58 (10.3%)	15/58 (25.9%)	0.001*	0.41
Adjusted OR (95% CI)^a^	Referent	5.69 (1.56–20.73)*	0.37 (0.06–2.26)	4.89 (1.32–18.14)*		

Mono-n-butyl phthalate						
Incidence of GDM (%)	13/66 (19.7%)	12/50 (24.0%)	9/58 (15.5%)	13/58 (22.4%)	0.26	0.81
Adjusted OR (95% CI)^a^	Referent	1.17 (0.40–3.41)	0.43 (0.13–1.44)	1.42 (0.52–3.88)		

Mono-(2-ethylhexyl) phthalate						
Incidence of GDM (%)	17/84 (20.2%)	8/32 (25.0%)	11/58 (19.0%)	11/58 (19.0%)	0.53	0.77
Adjusted OR (95% CI)^a^	Referent	2.14 (0.72–6.35)	1.14 (0.42–3.09)	1.03 (0.38–2.79)		

Mono-(2-ethyl-5-carboxypentyl) phthalate						
Incidence of GDM (%)	12/58 (20.7%)	11/58 (19.0%)	9/58 (15.5%)	15/58 (25.9%)	0.29	0.78
Adjusted OR (95% CI)^a^	Referent	0.61 (0.20–1.81)	0.42 (0.13–1.36)	1.19 (0.44–3.17)		

Mono(carboxyisooctyl) phthalate						
Incidence of GDM (%)	9/58 (15.5%)	13/58 (22.4%)	11/58 (19.0%)	14/58 (24.1%)	0.87	0.89
Adjusted OR (95% CI)^a^	Referent	1.54 (0.53–4.52)	1.18 (0.38–3.67)	1.39 (0.47–4.14)		

Bisphenol A						
Incidence of GDM (%)	11/57 (19.3%)	15/57 (26.3%)	13/57 (22.8%)	7/57 (12.3%)	0.07	0.24
Adjusted OR (95% CI)^a^	Referent	2.58 (0.84–7.94)	1.04 (0.31–3.53)	0.56 (0.14–2.28)		

Triclosan						
Incidence of GDM (%)	19/70 (27.1%)	8/44 (18.2%)	8/57 (14.0%)	11/57 (19.3%)	0.009*	0.022*
Adjusted OR (95% CI)^a^	Referent	0.25 (0.07–0.86)*	0.12 (0.03–0.55)*	0.35 (0.12–0.98)*		

Benzophenone-3						
Incidence of GDM (%)	10/68 (14.7%)	9/46 (19.6%)	16/57 (28.1%)	11/57 (19.3%)	0.83	0.95
Adjusted OR (95% CI)^a^	Referent	1.09 (0.38–3.14)	1.40 (0.52–3.77)	0.86 (0.29–2.51)		

*^a^Adjusted for age, pre-pregnancy body mass index (log-transformed), IMD (log-transformed), and parity; *n* = 197 for phthalates, *n* = 193 for phenols*.

*p < 0.05.

To explore the robustness of our results, and in view of the high incidence of GDM in our sample compared to previous studies (2), we repeated our analyses for MiBP and triclosan using the more stringent 1999 World Health Organization (WHO) criteria for GDM, i.e., fasting plasma glucose ≥7.0 mmol/l or 120-min plasma glucose ≥7.8 mmol/l (33). Using these criteria, the incidence of GDM was 27/232 (11.6%). The inverse association between incident GDM and triclosan persisted in both the continuous analysis (aOR 0.25, 95% CI 0.09–0.65, *p* = 0.005) and the categorical analysis (*p*_het_ = 0.28, *p*_trend_ = 0.006). However, there was no significant association between MiBP levels and incident GDM (data not shown).

Among women without GDM at 28 weeks of gestation (*n* = 185), MEHP was positively associated with 120-min plasma glucose (adjusted β 0.268, *p* = 0.0002), explaining 6.9% of the variation in 120-min glucose (Table S2 in Supplementary Material). MCiOP was also positively associated with 120-min glucose (adjusted β 0.183, *p* = 0.010), explaining 3.4% of the variation in 120-min glucose. Other phthalate metabolites (including MiBP) and all phenols were not significantly associated with fasting/stimulated glucose levels, HOMA2-B, HOMA2-IR, or disposition index (Table S2 in Supplementary Material).

Experimental studies suggest that MEHP, the primary metabolite of DEHP, may have greater endocrine disrupting activity that is secondary metabolites, such as MECPP (35). We, therefore, tested the associations between the molar ratio of MEHP to MECPP (MEHP/MECPP ratio), as an inverse marker of detoxification capability (23), and parameters of blood glucose homeostasis in women without GDM. The MEHP/MECPP ratio was positively associated with 120-min plasma glucose (adjusted β 0.280, *p* = 0.00008), explaining 7.6% of the variation in 120-min glucose, but not any other indices of glucose homeostasis.

We hypothesised that the lack of association of MEHP and MCiOP with GDM, despite their positive associations with 120-min plasma glucose, may reflect the relatively small proportion of cases in our sample with abnormal 120-min glucose levels, owing to the use of a higher diagnostic threshold (≥8.5 mmol/l) (31, 32) than has featured in previous GDM guidelines (e.g., ≥7.8 mmol/l) (33). We, therefore, repeated our GDM analyses for MEHP and MCiOP using the 1999 WHO criteria (33). In continuous models, neither MEHP (aOR per log increase in concentration 1.57, 95% CI 0.88–2.78, *p* = 0.12) nor MCiOP (aOR per log increase in concentration 3.02, 95% CI 0.81–11.26, *p* = 0.10) was associated with incident GDM; and MCiOP was not associated with GDM in the categorical analysis (*p*_het_ = 0.87, *p*_trend_ = 0.46). However, women with MEHP levels in the second (aOR 25.82, 95% CI 1.79–372.52, *p* = 0.017) and fourth (aOR 23.22, 95% CI 1.68–320.03, *p* = 0.019) quartiles, but not in the third quartile (aOR 8.09, 95% CI 0.59–111.44, *p* = 0.12), had significantly increased odds of GDM (*p*_het_ = 0.08, *p*_trend_ = 0.041).

Of the 232 male infants born to the women in our study, 16 (6.9%) had cryptorchidism at birth (Table 2). An additional 16 (6.9%) infant had cryptorchidism at 3, 12, or 24 months of age, but not at birth. Congenital cryptorchidism was not associated with maternal GDM (aOR 1.46, 95% CI 0.39–5.50, *p* = 0.58) or fasting/stimulated glucose levels, HOMA2-B, HOMA2-IR, or disposition index (data not shown). When we remodelled the of odds of GDM in relation to serum phthalate/phenol concentrations in the 216 mothers whose sons did not have cryptorchidism at birth, our results were not substantially different from the primary analyses: triclosan was associated with incident GDM in both the continuous analysis (aOR per log increase in concentration 0.48, 95% CI 0.28–0.82, *p* = 0.007) and categorical analysis (*p*_het_ = 0.027, *p*_trend_ = 0.019), and women with MiBP levels in the second (aOR 7.33, 95% CI 1.71–31.34, *p* = 0.007) and fourth (aOR 5.31, 95% CI 1.25–22.53, *p* = 0.024) quartiles, but not in the third quartile, had higher odds of GDM (*p*_het_ = 0.002, *p*_trend_ = 0.39) (other data not shown).

## Discussion

Using a prospective cohort design, we examined the associations between serum levels of phthalate metabolites, BPA, triclosan, and BP-3 measured at 10–17 weeks of gestation with GDM and indices of blood glucose homeostasis at 28 weeks of gestation. We observed a moderate inverse association between triclosan levels and GDM, and an apparent non-linear association between MiBP and GDM. We also demonstrated that MEHP, MCiOP, and the MEHP/MECPP ratio were positively associated with 120-min glucose levels but no other markers of glucose homeostasis in women without GDM. Sensitivity analyses using the 1999 WHO criteria for GDM suggested an additional non-linear association between MEHP levels and GDM. We did not find any significant associations for BPA.

Triclosan was inversely associated with incident GDM, with no evidence of a threshold or non-linear relationship. This unexpected association persisted in a sensitivity analysis using alternative GDM diagnostic criteria and a further analysis in which mothers whose sons had congenital cryptorchidism were excluded. There were no concomitant associations between triclosan and markers of glucose homeostasis in non-diabetic women. The only relevant experimental study of triclosan to date showed that administration to pregnant mice at 8 mg/kg/day—several thousand times the average human intake (36)—resulted in IGT with increased insulin resistance and reduced β-cell function at gestational day 17, although this effect was not seen at lower doses (1–4 mg/kg/day) (37). An epidemiological study found no associations between urinary triclosan levels and patient-reported diabetes, fasting glucose, or fasting insulin in 1,455 non-pregnant adults (20). However, a reduction in GDM incidence could, at least in part, explain a reported inverse association between maternal triclosan levels and fetal growth in the third trimester (38). Triclosan is a broad-spectrum antimicrobial agent commonly used in personal care products such as soaps, toothpastes, and deodorants (although in 2016, it was banned from hand/body washes by the US Food and Drug Administration and the European Commission) (39). The mechanism for its apparent protective effect against GDM is unknown, but possibilities might include a reduction in systemic inflammation, and consequently insulin resistance, through its antimicrobial and/or anti-inflammatory actions (40, 41), or an increase in catabolism and weight loss through mitochondrial uncoupling (41). Alternatively, the strong inverse association observed between triclosan and GDM risk may reflect residual confounding due to unmeasured maternal factors: for example, triclosan levels may be indicative of health-related behaviours such as better personal care and greater health consciousness, which may in turn be protective against GDM (2).

Of the phthalate metabolites, only MiBP was associated with incident GDM in our primary analyses, although in a non-linear manner, with increased odds in the second and top quartiles only. Non-monotonic dose–response relationships are well described in the EDC literature, including for certain phthalates (42); however, the majority of such relationships are biphasic. We could not find any historical data to support a possible mechanistic explanation for the *M*-shaped relationship observed for MiBP. Furthermore, MiBP was not associated with any other markers of glucose homeostasis in our study. We, therefore, conclude that our data do not support a true effect of MiBP on GDM.

Mono-(2-ethylhexyl) phthalate and MCiOP were positively associated with 120-min plasma glucose. These findings are consistent with the *in vitro* actions of phthalates (4) and rodent studies of DEHP (6). Two epidemiological studies of summed DEHP metabolites (including MEHP) have demonstrated associations with T2DM (7, 9)—although other studies assessing MEHP in isolation have not found associations with T2DM or 120-min glucose (7, 8), and none have assessed glycaemia in relation to MCiOP. We also found that the MEHP/MECPP ratio was positively associated with 120-min glucose, consistent with MEHP having greater endocrine disrupting activity than its secondary metabolites (35). The lack of association of these phthalate metabolites with fasting glucose, HOMA2-B, and HOMA2-IR could be due to a greater effect on stimulated, rather than baseline, insulin secretion, although we did not detect an association with 60-min insulin secretion (disposition index), either. We hypothesised that the lack of association with GDM in our primary analysis reflected the relatively small number of cases with abnormal 120-min glucose levels, and in a sensitivity analysis using a lower diagnostic threshold (≥7.8 mmol/l rather than ≥8.5 mmol/l), we found some evidence for an association between MEHP and incident GDM (significantly increased odds of GDM in the second and top quartiles only). It is possible that this association might be stronger in a population with higher MEHP levels, resulting in a greater incidence of abnormal 120-min glucose levels. In support of this, an association between summed DEHP metabolites and incident T2DM was reported in a cohort of women with high urinary concentrations (median 302–325 nmol/l) but not in a cohort with lower concentrations (median 230–278 nmol/l) (9).

Our phthalate results do not corroborate with three other recent prospective studies of GDM, which variously reported inverse associations of MiBP and MBzP with 60-min glucose (10), a positive association of MEP with IGT but an inverse association of DEHP metabolites with IGT (11), and no associations of any phthalates with IGT/GDM (12). Furthermore, we did not find any associations between BPA and incident GDM or markers of glucose homeostasis, in contrast to various epidemiological studies of BPA and T2DM (9, 15, 17), and one previous study (18), but not two others (12, 19), of BPA and gestational glycaemia. These discrepancies may reflect differences in methodology, such as the matrix of measurement (urine vs. serum) (43), glucose load used (50 vs. 75 g), or gestation at phthalate/BPA measurement (first vs. second trimester) (18); or differences between the study populations and our own: for example, the three cohorts in which relationships between phthalate levels and GDM were previously assessed had higher prevalences of overweight/obesity (>50, 45.4, and 37.3 vs. 33.8%) and non-white ethnicity (47.2, 41.1, and 15.5 vs. 1.7%) than our own; and our cohort did not include mothers of female infants.

The prevalence of GDM in our subcohort (20.3%) was several-fold higher than the reported prevalence for England and Wales (~3.5%) (1). This may reflect our use of the new international criteria for GDM (31, 32), which differ from those used by most previous population studies (2), and from those currently advocated in England and Wales (1); indeed, using older criteria (33), the prevalence in our subcohort was markedly lower (11.6%), although still higher than reported by most previous European population studies (0.6–22.3%) (2). Additionally, the women in our study may not be truly representative of the entire CBGS cohort. For example, several studies have suggested that women carrying a male fetus have an increased risk of GDM (44–46), and some authors have reported an association between GDM and congenital cryptorchidism (34), although we and others have found no such link (47).

We did not have data for all possible confounders: for example, education, income, activity levels, diet, subfertility, family history of diabetes, and other EDCs (12, 18). This may have led to overestimation of effect sizes, as may have any antagonistic interactions between EDCs. Conversely, we cannot rule out over-adjustment in our analyses: for example, if an EDC had independent effects on both adiposity and glucose intolerance, then, correction for BMI would have led to an underestimation of its effect on GDM.

Serum phthalate metabolite levels in our study were of a similar order of magnitude to those reported in other cohorts of pregnant or postpartum women (48, 49). Serum triclosan levels were ~10-fold to 20-fold lower than reported in Australian women of childbearing age (50), but of a similar range to those in US adults and children (51, 52).

Strengths of our study include its prospective design; use of laboratory criteria to diagnose GDM; sample collection and storage in glass tubes (to avoid phthalate/BPA contamination); use of a highly sensitive technique for measuring the chemicals; and our ability to account for a number of potential confounding variables. This is also the first study to examine the association between phthalate metabolites and phenols and markers of insulin resistance and β-cell function in pregnant women.

Our study has several limitations. First, the sample size was relatively small, which will have limited precision and statistical power, although it was larger than two of the five previously published studies of phthalates/BPA and GDM (10, 19). Second, our use of the 60-min disposition index to assess insulin secretion may have weakened associations between chemical levels and first-phase insulin secretion because the 60-min insulinogenic index is not as robust a measure of β-cell function as the 30-min insulinogenic index (53). Third, measurement of serum levels of the chemicals may have resulted in exposure misclassification: phthalates (24), BPA (54), and triclosan (55) all have short biological half-lives (<24 h), meaning that serum measurements may reflect recent brief exposures rather than the subjects’ daily habits; and the levels of these chemicals can be several orders of magnitude lower in serum than urine (24, 43), increasing the possibility that external/iatrogenic contamination has obscured true exposures. However, we believe that our positive findings are valid, for several reasons: we took steps to avoid external contamination of serum; MCiOP and MECPP are downstream phthalate metabolites, which are not known to form as a result of external contamination (43); and it is most likely that exposure misclassification would be non-differential, which would attenuate, rather than enhance, any relationships found. The final study limitation was that we examined effects of the phthalate metabolites and phenols individually, with no allowance for mixture effects or corrections for multiple testing. However, we feel that the latter is justified by the previously demonstrated associations with glucose metabolism for many of the chemicals (7–11, 15–17); and indeed most significant *p* values were ≤ 0.01, making a type-1 error unlikely.

In conclusion, our study provides a novel and interesting demonstration of inverse association between maternal serum triclosan levels at 10–17 weeks of gestation and incident GDM, in addition to biologically plausible associations between MEHP, MCiOP, and the MEHP/MECPP ratio and stimulated blood glucose levels. However, these findings need to be confirmed by further carefully designed prospective studies in other settings, that use a variety of specimen types (including urine) and assess exposure levels at multiple time points. More evidence is required before specific recommendations can be made with regards to clinical practice in diabetes or public health policy.

## Ethics Statement

This study was carried out in accordance with the recommendations of Cambridge Local Research Ethics Committee with written informed consent from all subjects. All subjects gave written informed consent in accordance with the Declaration of Helsinki. The protocol was approved by Cambridge Local Research Ethics Committee.

## Author Contributions

BF, AT, and CA contributed to the conception and design of the study; BF, HF, A-MA, AJ, and AT performed the data analysis; KO provided statistical expertise; BF wrote the first draft of the manuscript; AT, IH, DD, KO, and CA contributed to the design of the CBGS; and HF, A-MA, AJ, IH, DD, KO, and CA contributed to data collection. All authors contributed to the interpretation of the results and revision of the manuscript for important intellectual content, and approved the final version of the manuscript.

## Conflict of Interest Statement

The authors declare that the research was conducted in the absence of any commercial or financial relationships that could be construed as a potential conflict of interest.

## Abbreviations

BP-3, benzophenone-3; BPA, bisphenol A; CBGS, Cambridge Baby Growth Study; DEHP, di-(2-ethylhexyl) phthalate; EDC, endocrine disrupting chemical; GDM, gestational diabetes mellitus; HOMA2, updated homeostasis model assessment; IGT, impaired glucose tolerance; IMD, Index of Multiple Deprivation 2007; LOD, limit of detection; MBzP, monobenzyl phthalate; MCiOP, mono(carboxyisooctyl) phthalate; MECPP, mono-(2-ethyl-5-carboxypentyl) phthalate; MEHP, mono-(2-ethylhexyl) phthalate; MEP, monoethyl phthalate; MiBP, monoisobutyl phthalate; MnBP, mono-n-butyl phthalate; OGTT, oral glucose tolerance test; T2DM, type 2 diabetes mellitus.
