# Supplementary material for: Serum Phthalate and Triclosan Levels Have Opposing Associations With Risk Factors for Gestational Diabetes Mellitus
Source: Front Endocrinol (Lausanne). 2018 Mar 13;9:99. doi: 10.3389/fendo.2018.00099 (PMC5859030; doi:10.3389/fendo.2018.00099)
Supplement: Supplementary file 1 [file Data_Sheet_1.docx]

***Supplementary Material***

**Serum Concentrations of Phthalates and Phenols Are Associated With Risk of Gestational Diabetes Mellitus**

**Benjamin G. Fisher, Hanne Frederiksen, Anna-Maria Andersson, Anders Juul, Ajay Thankamony, Ken K. Ong, David B. Dunger, Ieuan A. Hughes, Carlo L. Acerini^*^**

**^*^ Correspondence**: Dr Carlo L. Acerini: cla22@cam.ac.uk

**Supplementary methods**

Serum samples were analysed for the total content of 16 phthalate metabolites [mono-ethyl phthalate (MEP), mono-isobutyl phthalate (MiBP), mono-n-butyl phthalate (MnBP), MBzP, mono-pentyl phthalate (MPP), mono-(2-ethylhexyl) phthalate (MEHP), mono-(2-ethyl-5-hydroxyhexyl) phthalate (MEHHP), mono-(2-ethyl-5-oxohexyl) phthalate (MEOHP), mono-(2-ethyl-5-carboxypentyl) phthalate (MECPP), mono-n-octyl phthalate (MOP), mono-(3-carboxypropyl) phthalate (MCPP), mono-isononyl phthalate (MiNP), mono(hydroxyisononyl) phthalate (MHiNP), mono-oxoisononyl phthalate (MOiNP), mono(carboxyisooctyl) phthalate (MCiOP), mono-isodecyl phthalate (MiDP)] and 9 phenols [bisphenol A (BPA), triclosan (TCS), triclocarban (TCC), benzophenone-3 (BP-3), 2,4-dichlorophenol (2,4-DCP), 2,5-dichlorophenol (2,5-DCP), 2,4,5-trichlorophenol (2,4,5-TCP), 2-phenylphenol (2-PP), and 4-phenylphenol (4-PP)] by isotope-diluted liquid chromatography coupled to tandem mass spectrometry, with preceding enzymatic deconjugation followed by solid phase extraction. The method for preparation of samples, standard solutions and quality controls, as well as the instrumental analysis and general method validation, was as previously described (1, 2). In the present study samples were analysed in eight batches during a period of five weeks (phthalate metabolites) and three weeks (phenols). In short, each batch included standards for calibration curves, approximately 30-40 unknown samples, two blanks, two serum pool controls, and two serum pool controls spiked with standards at low level (1.0 µg/l). The inter-/intra-day variation for phthalate metabolites, expressed as the relative standard deviation (RSD), was below 13% for most of the analytes, except MOP (20%), MiDP (17%), and MiDP (31%). The recovery of spiked control samples was above 90% for all phthalate analytes except MnBP, MPP, MOP, and MiNP (all >82%). The inter-/intra-day variation for phenols, expressed as the RSD, was ≤20% for all analytes. The recovery of spiked control samples was >90% for all phenol analytes, except TCC (83%). The limit of detection (LOD) was determined as previously described (1, 2). The fact that some subjects showed undetectable levels rules out a general contamination of these compounds. The measured levels of the phthalate monoesters (MEP, MiBP, MnBP, MBzP, MPP, MOP, MEHP, MiNP, MiDP) were considered to be semi-quantitative, as hydrolysis of potentially contaminating diester phthalates to monoester phthalates (e.g. DEHP to MEHP) by enzymes present in the blood may have occurred after the samples were drawn. However, the large inter-individual variation observed for these metabolites suggests that a systematic contamination of the samples with one or more of the phthalate diesters is unlikely. The measured levels for downstream phthalate metabolites (MEHHP, MEOHP, MECPP, MCPP, MHiNP, MOiNP, MCiOP) were quantitative, as conversion to these metabolites can occur only *in vivo*.

1. Frederiksen H, Jørgensen N, Andersson A-M. Correlations between phthalate metabolites in urine, serum, and seminal plasma from young Danish men determined by isotope dilution liquid chromatography tandem mass spectrometry. *J Anal Toxicol* (2010) **34**:400–410. doi:10.1038/jes.2010.6
2. Frederiksen H, Aksglaede L, Sorensen K, Nielsen O, Main KM, Skakkebaek NE, Juul A, Andersson A-M. Bisphenol A and other phenols in urine from Danish children and adolescents analyzed by isotope diluted TurboFlow-LC-MS/MS. *Int J Hyg Environ* *Health* (2013) **216**:710–20. doi:10.1016/j.ijheh.2013.01.007

**Supplementary tables**

Supplementary Table 1. Spearman correlations between serum concentrations of the 6 phthalate metabolites and 3 phenols that were measured above the LOD in >60% of samples (n=232 for phthalates, n=228 for phenols).

| **Analyte** | **MEP** | **MiBP** | **MnBP** | **MEHP** | **MECPP** | **MCiOP** | **BPA** | **Triclosan** | **BP-3** |
| --- | --- | --- | --- | --- | --- | --- | --- | --- | --- |
| MEP | 1.000 |  |  |  |  |  |  |  |  |
| MiBP | 0.165^*^ | 1.000 |  |  |  |  |  |  |  |
| MnBP | 0.133^*^ | 0.838^**^ | 1.000 |  |  |  |  |  |  |
| MEHP | -0.018 | 0.103 | 0.123 | 1.000 |  |  |  |  |  |
| MECPP | 0.123 | 0.093 | 0.061 | -0.016 | 1.000 |  |  |  |  |
| MCiOP | 0.051 | -0.019 | -0.029 | 0.158^*^ | 0.364^**^ | 1.000 |  |  |  |
| BPA | 0.079 | 0.092 | 0.105 | -0.140^*^ | 0.061 | -0.048 | 1.000 |  |  |
| Triclosan | 0.078 | -0.100 | -0.035 | -0.142^*^ | 0.041 | -0.121 | 0.136^*^ | 1.000 |  |
| BP-3 | 0.007 | -0.002 | -0.035 | -0.066 | 0.019 | -0.085 | -0.009 | 0.323 | 1.000 |

^*^ p<0.05.

^**^ p<0.0005.

Supplementary Table 2. Association between log-transformed serum concentrations of phthalate metabolites and phenols and parameters of glucose homeostasis in women without GDM.

| **Chemical** | **Log_10_(HOMA2-B)^a,b^** | | **Log_10_(HOMA2-IR)^a,c^** | | **Fasting plasma glucose^a,d^** | | **120-min plasma glucose^a,d^** | | **Log_10_(disposition index)^a,c^** | |
| --- | --- | --- | --- | --- | --- | --- | --- | --- | --- | --- |
|  | **β** | **P value** | **β** | **P value** | **β** | **P value** | **β** | **P value** | **β** | **P value** |
| MEP | 0.029 | 0.74 | -0.011 | 0.89 | -0.038 | 0.60 | 0.056 | 0.44 | -0.004 | 0.97 |
| MiBP | 0.007 | 0.94 | 0.024 | 0.75 | -0.047 | 0.51 | 0.059 | 0.41 | 0.018 | 0.84 |
| MnBP | 0.022 | 0.79 | 0.016 | 0.84 | -0.011 | 0.88 | 0.059 | 0.41 | 0.037 | 0.69 |
| MEHP | -0.011 | 0.89 | -0.118 | 0.13 | -0.053 | 0.46 | 0.268 | 0.0002^*^ | 0.014 | 0.88 |
| MECPP | -0.072 | 0.39 | -0.034 | 0.66 | -0.009 | 0.90 | -0.098 | 0.17 | 0.132 | 0.14 |
| MCiOP | -0.119 | 0.16 | 0.060 | 0.43 | 0.117 | 0.10 | 0.183 | 0.010^*^ | -0.041 | 0.65 |
| BPA | -0.050 | 0.56 | -0.061 | 0.43 | -0.040 | 0.58 | 0.049 | 0.50 | 0.073 | 0.42 |
| Triclosan | 0.071 | 0.41 | 0.013 | 0.87 | -0.035 | 0.63 | -0.096 | 0.19 | 0.005 | 0.96 |
| BP-3 | 0.169 | 0.051 | 0.006 | 0.93 | -0.106 | 0.15 | -0.019 | 0.80 | 0.009 | 0.92 |

^a^ Adjusted for age, pre-pregnancy BMI (log-transformed), IMD (log-transformed), and parity.

^b^ n=126 for phthalates, n=124 for phenols.

^c^ n=127 for phthalates, n=125 for phenols.

^d^ n=185 for phthalates, n=182 for phenols.

^e^ n=120 for phthalates, n=118 for phenols.

^*^ p<0.05.
